# Serum 17β-Estradiol Concentrations in Women With Interstitial Cystitis/Bladder Pain Syndrome: A Retrospective Observational Study

**DOI:** 10.7759/cureus.114021

**Published:** 2026-08-05

**Authors:** Tiberiu A Priporeanu, Ion Petre, Ramona E Dragomir, Robert I Stoica, Valentin M Pirvut, Nicolae Grigore

**Affiliations:** 1 Doctoral School, Lucian Blaga University of Sibiu, Sibiu, ROU; 2 Functional Science, Medical Informatics, and Biostatistics, Victor Babes University of Medicine and Pharmacy Timisoara, Timisoara, ROU; 3 Obstetrics-Gynecology, National Institute for Mother and Child Health "Alessandrescu-Rusescu", Bucharest, ROU; 4 Urology, Carol Davila University of Medicine and Pharmacy, Bucharest, ROU; 5 Urology, Lucian Blaga University of Sibiu, Sibiu, ROU

**Keywords:** bladder pain syndrome, estradiol serum levels, interstitial cystitis, menopause, pelvic pain

## Abstract

Introduction: Interstitial cystitis/bladder pain syndrome (IC/BPS) is a long-lasting, sterile inflammatory disease affecting the lower urinary tract, marked by pelvic pain, urgency, and nighttime urination (nocturia). The marked female predominance of IC/BPS has prompted interest in the potential role of endocrine factors in disease susceptibility and symptom variability. However, the relationship between circulating estrogen concentrations and IC/BPS remains incompletely understood, and clinical evidence has been inconsistent. This study aimed to characterize the baseline distribution of serum 17β-estradiol concentrations among women diagnosed with IC/BPS.

Methodology: We conducted a retrospective, observational, single-center study of 195 women with a confirmed diagnosis of IC/BPS according to European Association of Urology (EAU) and American Urological Association (AUA) criteria. Serum 17β-estradiol concentrations obtained during the initial clinical evaluation were retrieved from medical records. Hormone concentrations were categorized using established physiological reference intervals, and descriptive statistics summarized the distribution of serum estradiol levels.

Results: A total of 195 women were included in the study. Serum estradiol concentrations ranged from 3.1 to 712.7 pg/mL, with a median of 40.2 pg/mL and a mean of 68.7 ± 89.5 pg/mL. Overall, 109 patients (55.9%) had serum estradiol concentrations below 50 pg/mL, 43 (22.1%) between 51 and 100 pg/mL, 30 (15.4%) between 101 and 200 pg/mL, and 13 (6.6%) above 200 pg/mL.

Conclusions: Low serum 17β-estradiol concentrations were frequently observed in this cohort of women with IC/BPS. Although these findings are consistent with the hypothesis that endocrine factors may contribute to disease heterogeneity, the retrospective observational design and the absence of a healthy control group preclude conclusions about causality or clinical application. Prospective longitudinal studies incorporating standardized hormone assessment, appropriate control groups, and adjustment for potential confounding variables are needed to clarify the role of circulating estradiol in IC/BPS.

## Introduction

Interstitial cystitis/bladder pain syndrome (IC/BPS) is a chronic pain disorder marked by persistent pelvic pain, urinary urgency, frequency, and nocturia, occurring in the absence of urinary tract infection or other identifiable causes [1-3]. The condition substantially impairs quality of life and poses a significant clinical challenge because of its heterogeneous presentation and incompletely understood pathophysiology [1,2]. Epidemiological studies consistently show a marked female predominance, with women affected several times more often than men [4,5]. This sex disparity has generated increasing interest in the potential role of endocrine factors in disease susceptibility and symptom expression [6].

Current evidence suggests that IC/BPS is a multifactorial disorder involving alterations in urothelial barrier function, neurogenic inflammation, immune dysregulation, peripheral and central pain processing, and psychosocial factors [6,7]. Rather than a single disease entity, IC/BPS is increasingly regarded as a heterogeneous clinical syndrome in which several pathophysiological mechanisms may coexist [6-8]. Consequently, no single biological pathway has been established as the primary driver of disease development, and reliable biomarkers for diagnosis or disease stratification remain unavailable [6,9].

Experimental studies in animal models and in vitro systems suggest that estrogen signaling may influence several aspects of bladder physiology, including urothelial maintenance, inflammatory responses, and sensory function [10-14]. Estrogen receptors have been identified in the lower urinary tract, supporting the hypothesis that hormonal signaling may contribute to bladder homeostasis [10,11]. However, the biological relevance of these experimental observations in human IC/BPS remains incompletely understood. In particular, proposed interactions among estrogen receptor signaling, mast-cell activation, urothelial permeability, and nociceptive pathways have not been fully confirmed in clinical studies. These findings should therefore be interpreted cautiously [13-17].

Clinical observations also suggest that hormonal factors may influence symptom variability in some women with IC/BPS. Symptom exacerbation has been reported during periods of hormonal fluctuation, including the late luteal phase of the menstrual cycle and the menopausal transition [18,19]. Nevertheless, published clinical evidence has not established a consistent association between circulating estrogen concentrations and IC/BPS. Differences in study populations, menopausal status, timing of blood sampling, and study design may contribute to these heterogeneous observations. Consequently, the relationship between systemic estrogen status and IC/BPS remains uncertain and requires further investigation [6,19].

Although serum 17β-estradiol does not directly reflect estrogen activity in bladder tissue, it remains a widely available clinical marker of systemic estrogen status. Measuring circulating estradiol may therefore provide indirect information about endocrine status in women with IC/BPS and may generate hypotheses for future mechanistic and prospective clinical studies. However, serum estradiol concentrations are influenced by several physiological variables, including age, menopausal status, and menstrual cycle phase, which should be considered when interpreting hormonal measurements [20].

Despite growing interest in endocrine influences on IC/BPS, relatively few studies have described the baseline distribution of serum estradiol concentrations in women with clinically confirmed disease. Most previous investigations have focused on symptom fluctuations across reproductive stages or on potential biological mechanisms rather than on the hormonal profile of patients at initial clinical evaluation. Consequently, the prevalence of low circulating estradiol concentrations in IC/BPS cohorts remains poorly characterized.

The primary objective of this retrospective observational study was to characterize the baseline distribution of serum 17β-estradiol concentrations among women diagnosed with IC/BPS. Given the observational design, the study was intended to describe hormonal distributions and identify potential associations rather than establish causal relationships or confirm proposed biological mechanisms.

## Materials and methods

This retrospective, observational, single-center study was conducted to characterize the baseline distribution of serum 17β-estradiol concentrations among women diagnosed with IC/BPS. Given the study's descriptive nature and the absence of a healthy comparison group, the analysis was intended to describe the cohort's hormonal profile rather than establish causal relationships between estradiol concentrations and IC/BPS.

The study was based on data extracted from the medical records of patients evaluated between January 2020 and December 2025. Clinical and laboratory data were obtained from the available medical documentation. The study was conducted in accordance with the ethical principles of the Declaration of Helsinki and applicable data protection regulations and was approved by the institutional Ethics Committee.

Inclusion and exclusion criteria

Inclusion criteria mandated a confirmed diagnosis of IC/BPS by a specialist, following the current guidelines of the European Association of Urology (EAU) and the American Urological Association (AUA), based on the clinical triad [2]: chronic pelvic/suprapubic pain or discomfort (lasting at least six months and exacerbated by bladder filling), persistent urinary urgency, frequency/nocturia, and mandatory availability of the quantitative serum 17β-estradiol measurement from the initial evaluation in the database. Exclusion criteria included active pelvic oncological malignancies (bladder, cervical, endometrial, or ovarian cancer), a history of pelvic radiation therapy (radiation cystitis) or recent systemic oncological treatments (such as cyclophosphamide chemotherapy), and documented current use (within the past three months) of systemic or topical hormone replacement therapy (HRT) or combined/progestin-only hormonal contraception, to prevent distortion of endogenous estradiol values.

Data collection

The study sample consisted of a consecutive cohort of 195 female patients who were evaluated, diagnosed, and monitored for IC/BPS. Clinical and demographic data were retrospectively collected through a comprehensive review of medical charts (observation files) and patient anamneses, along with corresponding laboratory test results. Because this investigation was retrospective, blood samples had been collected during routine clinical care rather than according to a standardized research protocol. Consequently, the timing of blood sampling relative to the menstrual cycle was not standardized.

For a detailed statistical and clinical analysis, the cohort of 195 patients was stratified into four subgroups of interest, based on plasma estradiol concentration (Table 1) [20].

**Table 1:** Estradiol plasma concentration categories. Plasma concentrations of estradiol (17β-estradiol) were grouped into four intervals to characterize the cohort status. The lower concentration interval (50 pg/mL) may reflect postmenopausal status or premature ovarian insufficiency. The low-intermediate concentration interval (51-100 pg/mL) corresponds to perimenopause or the mid-follicular phase. The intermediate concentration interval (101-200 pg/mL) represents an optimal luteal phase for women of reproductive age, while the higher concentration interval (200 pg/mL) captures the periovulatory surge or peripheral aromatization [20].

Estradiol plasma concentration	Descriptive category
0-50 pg/mL	Lower concentration interval
51-100 pg/mL	Low-intermediate concentration interval
101-200 pg/mL	Intermediate concentration interval
Above 200 pg/mL	Higher concentration interval

Statistical analysis was performed using JASP version 0.18. JASP is free, open-source software developed and maintained by the University of Amsterdam (UvA), Amsterdam, The Netherlands.

The distribution of continuous variables was evaluated using the Kolmogorov-Smirnov and Shapiro-Wilk tests. Because serum 17β-estradiol concentrations showed a non-normal distribution, continuous variables are presented as median, mean, standard deviation, minimum, and maximum values. Categorical variables are summarized as absolute frequencies and percentages. Given the descriptive objective of the study and the absence of a healthy comparison group, no inferential analyses comparing the study cohort with an external reference population were performed.

## Results

A total of 195 women with a confirmed diagnosis of IC/BPS were included in the study. Complete serum 17β-estradiol measurements obtained during the initial clinical evaluation were available for all participants; therefore, no missing laboratory data were identified for the primary study variable.

**Figure 1:**
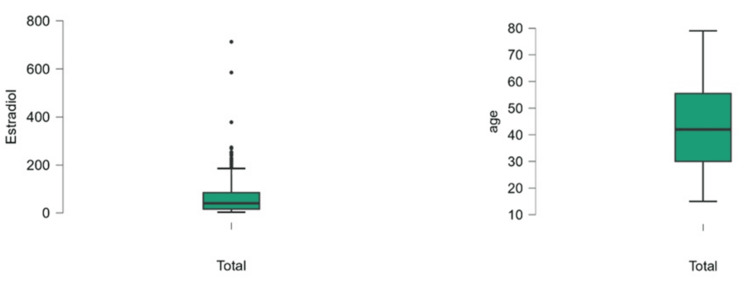
Estradiol levels and age in the study group. The figure presents the baseline descriptive statistics for the two primary parameters monitored in the study cohort. The first panel shows the serum estradiol (17β-estradiol) concentrations of the 195 patients with IC/BPS, and the second panel displays their age distribution. In each box, the horizontal line indicates the median, the box boundaries represent the interquartile range (IQR), and the whiskers illustrate the variability of the data and possible outliers. Image generated by the authors using Microsoft Office 2019 Excel and edited using Microsoft Paint (Microsoft Corporation, Redmond, WA). IC/BPS, interstitial cystitis/bladder pain syndrome

In the first stage, we analyzed two patient parameters: estradiol levels and age, which are presented as boxplots (Figure 1).

The summary statistics are presented in Table 2.

**Table 2:** Summary statistics for estradiol levels and age. The table presents baseline statistics for the primary variables in the study cohort (*N* = 195). Serum estradiol (17β-estradiol) levels are reported in picograms per milliliter (pg/mL), and patient age is reported in years. Mean, arithmetic average; standard error, standard error of the mean; median, 50th percentile; standard deviation, measure of data dispersion; range, interval between the minimum and maximum values; count, total number of patients

Summary statistics	Estradiol (pg/mL)	Age (years)
Mean	69	44
Standard error	6.4	1.1
Median	40.2	42.0
Standard deviation	89.5	14.8
Range	709.6	64.0
Minimum	3.1	15.0
Maximum	712.7	79.0
Count	195	195

The study participants' ages ranged from 15 to 79 years, with a mean age of 43.8 years. The distribution of patients across the specific age groups analyzed is represented in Table 3.

**Table 3:** Age distribution. The table presents the demographic breakdown of the study cohort (*N* = 195) into three age brackets reflecting distinct reproductive and endocrine phases: young reproductive age (under 39 years), perimenopausal transition (40-45 years), and postmenopausal or advanced age (over 45 years). All age values are expressed in years. Valid, number of patients per subgroup; mean, arithmetic mean age within each bracket; minimum/maximum, recorded age boundaries for each group

	Under 39 years old	40-45 years old	Over 45 years old
Valid	80	34	81
Mean	30	42	59
Minimum	15	40	46
Maximum	39	45	79

Serum 17β-estradiol concentrations demonstrated substantial interindividual variability, ranging from 3.1 to 712.7 pg/mL. Assessment of data distribution using the Kolmogorov-Smirnov and Shapiro-Wilk tests indicated a non-normal distribution. Accordingly, descriptive statistics are presented primarily using the median together with the mean and standard deviation. The median serum estradiol concentration was 40.2 pg/mL, whereas the mean concentration was 68.7 ± 89.5 pg/mL. The marked difference between the median and the mean, together with the wide range of observed values, is consistent with a right-skewed distribution of serum estradiol concentrations.

For descriptive purposes, serum estradiol concentrations were categorized according to predefined physiological reference intervals [20]:

- Low values (below 50 pg/mL): expected at menopause, before puberty, or in ovarian dysfunction

- Low-intermediate values ( 51 - 100 pg/mL): specific to young (reproductive) women, depending on the phase of the cycle

- Intermediate values (101-200 pg/mL): can occur during ovulation, pregnancy, or in vitro fertilization (IVF) treatments

- High values (above 200 pg/mL): may be associated with ovarian stimulation, ovarian tumors, or obesity (estrogen is stored in adipose tissue)

**Figure 2:**
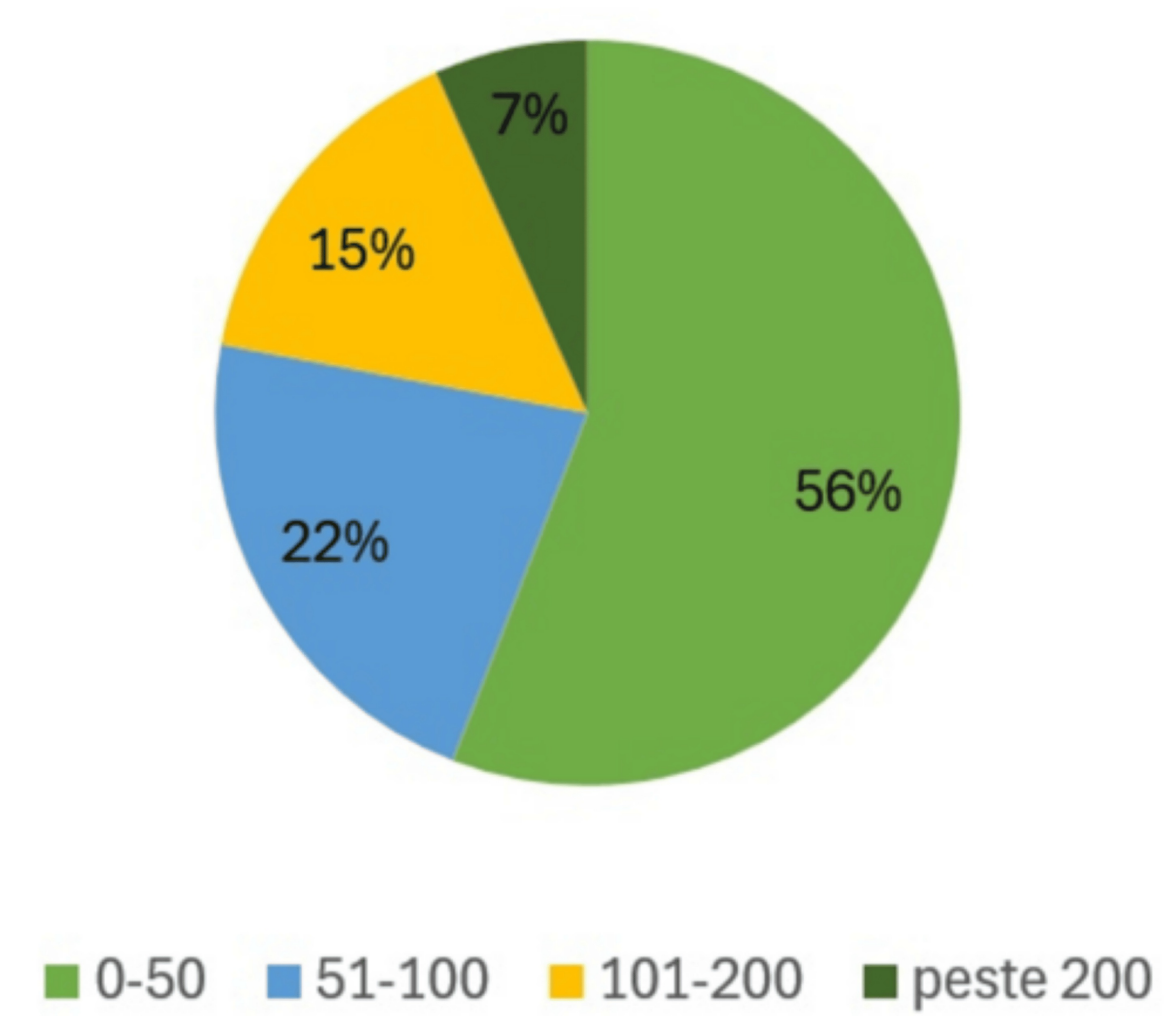
Percentage distribution of serum estradiol levels. This pie chart shows the relative percentage distribution of the 195 patients diagnosed with interstitial cystitis/bladder pain syndrome (IC/BPS) across specific serum estradiol (17β-estradiol) ranges. The segments highlight a clear dominance of severe hypoestrogenism, with 56% (exact: 55.9%) of the cohort presenting with hormone levels between 0 and 50 pg/mL. The remaining patient distribution spans 51-100 pg/mL (22%), 101-200 pg/mL (15%), and >200 pg/mL (7%). Image generated by the authors using Microsoft Office 2019 Excel and edited using Microsoft Paint (Microsoft Corporation, Redmond, WA).

The results are presented in Table 4 and Figure 2.

**Table 4:** Distribution of serum estradiol levels in the study group. The table presents the numerical distribution (*N* = 195) of the interstitial cystitis/bladder pain syndrome (IC/BPS) cohort, stratified by specific plasma estradiol (17β-estradiol) concentration ranges.

Values of the estradiol interval	*n* (%)
0-50	109 (55.9%)
51-100	43 (22.1%)
101-200	30 (15.4%)
Above 200	13 (6.6%)

It was observed that more than half of the patients in the study (109, 55.9%) had estradiol levels below 50 pg/mL, indicating a clear dominance of hypoestrogenism in the analyzed cohort. In contrast, only a small proportion of patients (13, 6.6%) had estradiol levels exceeding 200 pg/mL.

## Discussion

The present retrospective observational study characterized the baseline distribution of serum 17β-estradiol concentrations in a cohort of women with clinically confirmed IC/BPS. More than half of the patients (109, 55.9%) presented serum estradiol concentrations below 50 pg/mL at the time of initial evaluation, while only 13 (6.6%) exhibited concentrations exceeding 200 pg/mL. These findings indicate that low circulating estradiol concentrations were common within this study population. However, because the study was descriptive and retrospective and did not include a healthy comparison group, the observed hormonal distribution should be interpreted as a characteristic of this cohort rather than as evidence of a causal relationship between estradiol deficiency and IC/BPS.

The predominance of low serum estradiol concentrations observed in the present study is broadly consistent with previous clinical observations suggesting that hormonal status may influence symptom expression in some women with IC/BPS. Worsening bladder pain, urinary urgency, and urinary frequency have been reported during periods of physiological hormonal fluctuation, including the late luteal phase of the menstrual cycle [18].

**Figure 3:**
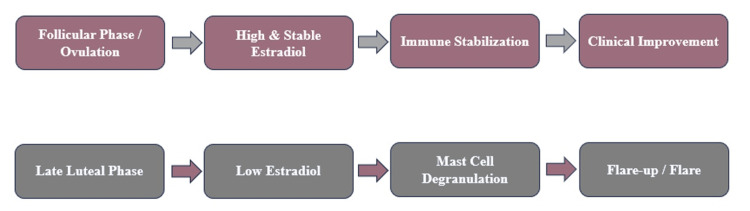
Relationship between estradiol levels and menstrual cycle phases. This flowchart maps the distinct neuroendocrine mechanisms that dictate symptom variation during the fertile period in patients with IC/BPS. The top cascade shows how the follicular phase and ovulation maintain high, stable estradiol levels, thereby stabilizing the local immune response within the bladder wall and leading to subsequent clinical improvement. Conversely, the bottom cascade outlines the trigger in the late luteal phase, in which a sudden drop to low estradiol levels induces acute bladder mast cell degranulation, precipitating a clinical flare-up. Image created by the authors using Microsoft Office 2019 PowerPoint and edited using Microsoft Paint (Microsoft Corporation, Redmond, WA). IC/BPS, interstitial cystitis/bladder pain syndrome

Possible mechanisms are presented in Figure 3.

Recent gynecologic consensus work also recognizes hormone-associated genitourinary changes as potentially relevant comorbid considerations in IC/BPS, while emphasizing the need for individualized clinical assessment [19]. Nevertheless, the available clinical evidence remains heterogeneous, and a consistent association between circulating estradiol concentrations and IC/BPS has not been established.

Differences among published studies may partly reflect substantial methodological variability. Previous investigations have included women of different reproductive ages, variable proportions of premenopausal and postmenopausal participants, heterogeneous diagnostic criteria, and non-standardized timing of hormone measurements. Because serum estradiol concentrations vary considerably according to menstrual cycle phase, menopausal status, ovarian function, and other physiological conditions, comparisons across studies remain challenging [20]. Consequently, the present findings should be interpreted within the broader context of clinical and pathological heterogeneity rather than as confirmation of a specific endocrine mechanism underlying IC/BPS [8,21-25].

An important observation in the present cohort is that not all women exhibited low serum estradiol concentrations. Approximately 44% of patients had concentrations above 50 pg/mL, including a small subgroup with values exceeding 200 pg/mL. This finding emphasizes the biological heterogeneity of IC/BPS and suggests that systemic estrogen status alone is unlikely to explain disease occurrence or symptom severity. Because information regarding menopausal status, body mass index, ovulatory status, endocrine disorders, and peripheral aromatization was not consistently available in this retrospective study, the reasons underlying higher estradiol concentrations in these patients cannot be determined. These findings instead support the concept that IC/BPS represents a heterogeneous clinical syndrome in which endocrine factors may contribute in only a subset of affected individuals.

The present study was designed to describe the hormonal profile of women with IC/BPS rather than to investigate the biological mechanisms linking estrogen signaling to bladder dysfunction. Consequently, the observed distribution of serum estradiol concentrations should be considered hypothesis-generating. While the findings are compatible with the possibility that endocrine factors contribute to disease heterogeneity, they do not demonstrate that low circulating estradiol concentrations are involved in disease initiation, progression, or symptom severity. Establishing such relationships will require prospective studies incorporating appropriate control populations, standardized hormonal assessment, longitudinal follow-up, and adjustment for relevant clinical confounders.

Several biological mechanisms have been proposed to explain how estrogen signaling might influence bladder physiology and potentially contribute to IC/BPS. Experimental studies conducted primarily in animal models and in vitro systems suggest that estrogen may help maintain urothelial homeostasis, modulate inflammatory responses, and influence sensory pathways within the lower urinary tract [10-17]. However, the clinical relevance of these experimental findings remains incompletely understood, and direct evidence in patients with IC/BPS is still limited [6,14,25].

One proposed mechanism involves the urothelial barrier. Experimental investigations have suggested that reduced estrogen signaling may be associated with alterations in urothelial permeability and glycosaminoglycan (GAG) metabolism, potentially facilitating exposure of suburothelial tissues to urinary solutes and inflammatory mediators [13-16]. Nevertheless, these mechanisms have been demonstrated predominantly in experimental models, and the present study did not evaluate urothelial integrity, GAG expression, or biomarkers of epithelial dysfunction. Consequently, no conclusions regarding these pathways can be drawn from the current data.

Similarly, previous studies have suggested that estrogen receptor signaling may influence inflammatory responses through interactions with immune cells, including mast cells [11,17,26-30]. Mast-cell activation has been implicated in the pathophysiology of IC/BPS, although its contribution appears to vary among clinical and pathological phenotypes [23,26-30]. Experimental evidence indicates that estrogen may modulate mast-cell activity under certain conditions; however, the underlying mechanisms remain incompletely understood, and consistent evidence in human IC/BPS is lacking. Because markers of mast-cell activation were not measured in the present study, our findings neither confirm nor refute the involvement of this pathway.

Experimental research has also proposed interactions between estrogen signaling and sensory pathways involving mediators such as nerve growth factor (NGF), transient receptor potential vanilloid-1 (TRPV1), and purinergic receptors [31,32]. These mechanisms have been suggested to contribute to nociceptive sensitization and chronic bladder pain in laboratory models. Reviews of overlapping urgency and sensory symptoms further emphasize that the neurobiological basis of IC/BPS remains incompletely resolved [33]. Oxidative stress and chronic inflammation have also been proposed as interacting pathways in IC/BPS, but their relationship with circulating estradiol was not examined in the present study [34]. Therefore, any relationship between serum estradiol concentrations and these pathways remains speculative within the context of this study.

Importantly, serum 17β-estradiol represents a measure of systemic endocrine status rather than local estrogen activity within bladder tissue. Circulating hormone concentrations do not necessarily reflect tissue-specific receptor expression, intracellular signaling, or local estrogen metabolism. Consequently, although low serum estradiol concentrations were frequently observed in the present cohort, these measurements should not be interpreted as direct evidence of altered estrogen signaling within the bladder. Future studies combining circulating hormone measurements with tissue biomarkers, receptor expression analyses, or molecular profiling may provide a more comprehensive understanding of the potential role of estrogen in IC/BPS. Also, therapeutic decisions should remain guided by clinical practice guidelines and intervention-specific evidence rather than by the observational findings reported here [1,22,35].

Several limitations should be considered when interpreting the present findings. First, the retrospective single-center design may have introduced selection bias and limits the generalizability of the results. Second, the absence of a healthy control group precludes determination of whether the observed distribution of serum estradiol concentrations differs from that of women without IC/BPS. Consequently, the present findings should be interpreted as describing the hormonal profile of this patient cohort rather than demonstrating an association between estradiol status and disease occurrence.

Third, serum 17β-estradiol concentrations were obtained from a single measurement performed during routine clinical evaluation. Because blood sampling was not standardized according to the menstrual cycle and repeated hormone measurements were unavailable, physiological fluctuations in circulating estradiol could not be accounted for. This limitation is particularly relevant for premenopausal women, in whom serum estradiol concentrations may vary substantially throughout the menstrual cycle.

Fourth, several potentially important confounding variables-including menopausal status, body mass index, endocrine disorders, ovarian function, metabolic status, smoking and other lifestyle characteristics-were not consistently available in the medical records and therefore could not be included in the analysis. Similarly, multivariable analyses and subgroup comparisons according to menopausal status could not be performed. These factors may have influenced the observed distribution of serum estradiol concentrations and should be systematically addressed in future investigations.

Despite its limitations, the present study has several strengths. It includes a relatively large cohort of women with clinically confirmed IC/BPS evaluated within a single tertiary referral center using standardized diagnostic criteria. In addition, serum 17β-estradiol concentrations were available for all included patients at the time of initial clinical evaluation, allowing a consistent description of baseline hormonal distribution within the study population. These data contribute to the limited clinical literature describing systemic estrogen status in women with IC/BPS and may serve as a basis for future hypothesis-driven investigations.

Prospective multicenter studies including age-matched healthy control groups are needed to determine whether the hormonal distribution observed in women with IC/BPS differs from that of the general female population. Future investigations should also standardize the timing of blood sampling according to menstrual cycle phase, incorporate repeated hormone measurements and systematically record menopausal status, body mass index, endocrine comorbidities, medication use and other potential confounding variables.

In addition, integrating endocrine assessment with validated symptom scores, bladder phenotyping, urinary and tissue biomarkers, and molecular analyses may help clarify whether specific hormonal profiles are associated with distinct clinical phenotypes of IC/BPS [8,9,23-25]. Such comprehensive approaches may improve understanding of disease heterogeneity and facilitate the identification of biologically meaningful patient subgroups. However, whether circulating estradiol has an independent role in disease susceptibility, symptom severity, or treatment response remains to be established.

## Conclusions

This retrospective observational study outlined the baseline serum 17β-estradiol levels in women with confirmed IC/BPS. Over half of the participants had serum estradiol levels below 50 pg/mL at initial evaluation, showing that low circulating estradiol is prevalent in this group.

Although these findings are consistent with the hypothesis that endocrine factors may contribute to the biological heterogeneity of IC/BPS, the present study does not establish a causal relationship between circulating estradiol concentrations and disease development or symptom severity. The absence of a healthy control group, the retrospective design, and the lack of adjustment for important confounding variables limit the interpretation of the observed hormonal distribution.

The present findings provide descriptive clinical data that may inform future research on endocrine influences in IC/BPS. Prospective longitudinal studies incorporating standardized hormone assessment, repeated measurements, appropriate control groups, and multivariable analyses are needed to clarify whether circulating estradiol has an independent role in disease susceptibility, clinical phenotype, or treatment response.
